# A Splice Form of VEGF, a Potential Anti-Angiogenetic Form of Head and Neck Squamous Cell Cancer Inhibition

**DOI:** 10.3390/ijms25168855

**Published:** 2024-08-14

**Authors:** Cristina Stefania Dumitru, Marius Raica

**Affiliations:** Department of Microscopic Morphology/Histology, Angiogenesis Research Center, “Victor Babes” University of Medicine and Pharmacy, 300041 Timisoara, Romania; marius.raica@umft.ro

**Keywords:** VEGF165b, head and neck squamous cell carcinoma, VEGF splice variants, tumor angiogenesis, VEGF splice form, anti-angiogenic therapy

## Abstract

Angiogenesis, primarily mediated by vascular endothelial growth factor (VEGF), is a fundamental step in the progression and metastasis of head and neck squamous cell carcinoma (HNSCC). Traditional anti-angiogenic therapies that target the *VEGF* pathway have shown promise but are often associated with significant side effects and variable efficacy due to the complexity of the angiogenic signaling pathway. This review highlights the potential of a specific *VEGF* splice form, *VEGF165b*, as an innovative therapeutic target for HNSCC. *VEGF165b*, unlike standard VEGF, is a natural inhibitor that binds to *VEGF* receptors without triggering pro-angiogenic signaling. Its distinct molecular structure and behavior suggest ways to modulate angiogenesis. This concept is particularly relevant when studying HNSCC, as introducing *VEGF165b’s* anti-angiogenic properties offers a novel approach to understanding and potentially influencing the disease’s dynamics. The review synthesizes experimental evidence suggesting the efficacy of VEGF165b in inhibiting tumor-induced angiogenesis and provides insight into a novel therapeutic strategy that could better manage HNSCC by selectively targeting aberrant vascular growth. This approach not only provides a potential pathway for more targeted and effective treatment options but also opens the door to a new paradigm in anti-angiogenic therapy with the possibility of reduced systemic toxicity. Our investigation is reshaping the future of HNSCC treatment by setting the stage for future research on VEGF splice variants as a tool for personalized medicine.

## 1. Introduction

Head and neck squamous cell carcinoma (HNSCC) is the sixth most common cancer worldwide, with approximately 600,000 new cases diagnosed annually. The mortality rate remains significant, with a five-year survival rate of around 50%, largely due to late-stage diagnosis and the aggressive nature of the disease. Major risk factors contributing to the development of HNSCC include tobacco use, excessive alcohol consumption, and infection with human papillomavirus (HPV). Given the high incidence and mortality rates, there is an urgent need for more effective therapeutic strategies, including those targeting angiogenesis [1].

Angiogenesis, the process of new blood vessel formation, is a critical event in the progression and metastasis of various cancers, including HNSCC [2]. Vascular endothelial growth factor (*VEGF*) is a key regulator of angiogenesis, promoting endothelial cell proliferation, migration, and vascular permeability. The discovery of *VEGF* and its role in angiogenesis revolutionized cancer research, leading to the development of anti-angiogenic therapies targeting *VEGF* pathways [3].

Despite advances in anti-angiogenic therapy, challenges remain in accurately assessing the efficacy of these treatments due to the lack of standardized criteria [4]. Furthermore, while *VEGF* expression is detected by immunohistochemistry (IHC) in both normal and tumor tissues, its behavior does not always correlate with new vessel formation, suggesting a more nuanced role in the regulation of angiogenesis. The role of biomarkers in cancer diagnosis and treatment is well recognized. For example, the detection of thymine dimers in renal cell carcinoma (RCC) using immunohistochemistry highlights the importance of identifying novel biomarkers for better clinical management and therapeutic targeting in different types of cancer, including HNSCC [5,6,7,8]. Interestingly, adding complexity to our understanding of *VEGF’s* role in angiogenesis regulation, certain splice forms of *VEGF* exhibit inhibitory properties. Among these splice variants, *VEGF165b* has received attention for its ability to block the pro-angiogenic effects of other *VEGF* isoforms by binding to *VEGF* receptors [9].

The discovery of *VEGF* as a key regulator of angiogenesis represents a defining moment in cancer research and therapy development. *VEGF* was first identified in the 1980s, with significant contributions from researchers such as Napoleone Ferrara, who isolated and cloned *VEGF* in 1989, revealing its important role in angiogenesis [10]. This discovery paved the way to understanding how tumors stimulate blood vessel formation to sustain their growth and spread, a process essential for tumor progression and metastasis [11]. *VEGF* acts by promoting endothelial cell proliferation, migration, and increased vascular permeability. Its role in angiogenesis has rapidly positioned *VEGF* as a prime target for therapeutic intervention in cancer treatment. The recognition of VEGF’s involvement in tumor angiogenesis has led to the development of anti-angiogenic therapies aimed at inhibiting the *VEGF* pathway to deprive tumors of blood supply, thus inhibiting their growth and metastatic potential [12]. Another important role of *VEGF* is in vascular endothelial development and optimal vascular endothelial function, and it is recognized for its role in the maturation of arterial and venous endothelium. This mechanism, essential in the physiological context, also extends to pathological conditions, particularly in the well-vascularized environment of malignant tumors. Given the importance of *VEGF*, its inhibitors used in oncological treatments justify prudent use, especially given their potential contribution to arterial aneurysm and dissection [13,14]. This phenomenon underscores the need for cardiovascular and oncology professionals to remain vigilant to this rare but serious risk, ensuring a balanced approach to *VEGF* inhibitor therapy in cancer patients [15].

In the evolution of cancer therapy, particularly in HNSCC, the promise of anti-angiogenic therapy has opened up new ways of treatment. Despite the theoretical attractiveness and preclinical success of anti-angiogenic therapies, a significant challenge in their clinical application is the lack of standardized criteria for evaluating their efficacy. This gap not only hinders the evaluation of current treatments but also hampers the development of future anti-angiogenic strategies [16,17].

Challenges in evaluating the efficacy of anti-angiogenic therapy:

1. Heterogeneous response: Tumors can vary greatly in their response to anti-angiogenic therapy, not only between different cancers but also between patients with the same cancer. This variability makes it difficult to establish universal criteria for efficacy and treatment [18].
2. The dynamic nature of tumor angiogenesis: Tumors can adapt to anti-angiogenic therapy over time, either by activating alternative angiogenic pathways or by adopting less angiogenesis-dependent mechanisms. This ability to adapt means that the efficacy of therapy may decrease with time or that initial responses may not be sustained, complicating the assessment of long-term efficacy [19].
3. Lack of direct biomarkers: There is a lack of direct, reliable biomarkers that can accurately reflect changes in angiogenesis due to therapy. Most current methods assess tumor size or growth rates using imaging techniques such as MRI (magnetic resonance imaging) or CT (computed tomography). However, these indicators may not sensitively or specifically reflect changes in angiogenesis, especially in the early stages of treatment [20].
4. Difficulty in measuring microenvironment changes: Anti-angiogenic therapies not only affect tumor cells, but also have a significant impact on the tumor microenvironment, including altering vascular permeability, interstitial pressure, and hypoxia. These changes are difficult to measure directly and quantitatively in patients [21].

The presence of *VEGF* in a wide range of normal and tumor tissues has been well documented by IHC, a vital technique for visualizing specific proteins in tissue sections. *VEGF*, as the main regulator of angiogenesis, is known to correlate with the formation of new blood vessels, which is a distinctive sign of tumor growth and metastasis. However, VEGF expression does not consistently determine angiogenesis, indicating a more complex role than simply as a direct facilitator of new vessel formation [22].

In the context of normal physiology, *VEGF* expression is essential for various functions, including wound healing and the menstrual cycle, where angiogenesis is a normal and controlled process. In these situations, *VEGF* expression is tightly regulated, and its effects are balanced by a suite of other angiogenic and anti-angiogenic factors that maintain vascular homeostasis [23]. In contrast, tumor tissues often show increased *VEGF* expression, which has been commonly associated with the promotion of angiogenesis within the tumor microenvironment. This angiogenic change is considered an essential step in the transition of tumors from a latent to a malignant, invasive condition. Increased levels of *VEGF* are expected to correspond with increased angiogenesis and, consequently, tumor progression [24].

*VEGF* achieves its pro-angiogenic effects by binding to specific receptors on the surface of endothelial cells, which leads to a cascade of signaling events that result in the proliferation, migration, and formation of new blood vessels by endothelial cells [25]. In addition to its known angiogenic role, *VEGF* also has naturally occurring inhibitory forms. These inhibitory forms of *VEGF* are splicing variants that come from the same *VEGF* gene but are processed differently, resulting in proteins that can inhibit the effects of the standard *VEGF* molecule. The most studied of these is *VEGF165b*, an isoform that competes with the angiogenic form of *VEGF* for binding to the receptor but does not activate the receptor signaling pathways that lead to angiogenesis. Instead, it inhibits these pathways, resulting in an anti-angiogenic effect [26].

Of the *VEGF* splice variants, *VEGF165b* has emerged as a molecule of interest because of its intrinsic anti-angiogenic properties. Unlike its pro-angiogenic homologs, *VEGF165b* does not stimulate vascular growth [27]; instead, it binds to VEGF receptors with similar affinity but without activating the angiogenic signaling cascade [28]. This unique feature of *VEGF165b* positions it as a novel and promising target for the inhibition of angiogenesis in the context of squamous cell carcinoma of the head and neck. The therapeutic potential of *VEGF165b* is based on its ability to naturally inhibit vascular proliferation, which is essential for HNSCC progression and metastasis [29]. Understanding this form of splicing could lead to the development of innovative anti-angiogenic strategies, potentially improving patient outcomes by preventing tumor growth and spread while minimizing the side effects associated with conventional anti-angiogenic drugs.

## 2. Understanding the Splice Forms of *VEGF*

### 2.1. The Biology of VEGF Splicing

Angiogenesis is indispensable for both physiological growth and pathological developments, such as tumorigenesis in HNSCC [17]. *VEGF*, an essential factor in angiogenic regulation, is synthesized by a finely regulated process known as alternative splicing. This post-transcriptional mechanism facilitates the generation of multiple *VEGF* isoforms from a single gene, enriching the VEGF family to include both the promotion and inhibition of angiogenesis [30].

**Figure 1:**
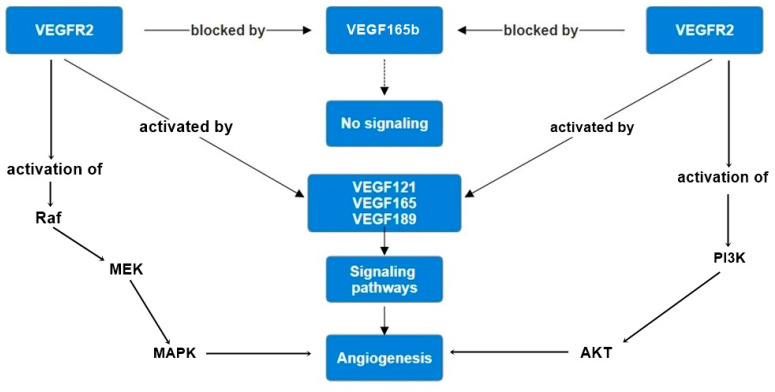
*VEGF* signaling pathways. *VEGF* binding to *VEGFR* activates several downstream signaling cascades, including the MAPK pathway (RAF-MEK-MAPK), leading to cell proliferation and survival, and the PI3K pathway (PI3K-AKT), promoting cell growth and survival. These pathways contribute to angiogenesis and are critical in the context of tumor progression.

The *VEGF* gene is alternatively spliced through its series of exons to produce different isoforms, each with different receptor-binding characteristics and subsequent biological activities. The pro-angiogenic isoforms—*VEGF121*, *VEGF165*, and *VEGF189*—are capable of binding to the *VEGF* receptors, *VEGFR1* and *VEGFR2*, on endothelial cells, thereby initiating a cascade of cellular events leading to proliferation, migration, and ultimately new vessel formation (Figure 1) [31].

These isoforms also have different affinities for heparin and heparan sulfate proteoglycans, determining their solubility and influence on the extracellular matrix, which in turn influences tissue-specific vascular growth patterns [32].

In contrast, *VEGF165b*, together with the b-series *VEGFs*, represents the anti-angiogenic faction of the *VEGF* family. This variant results in a protein with a divergent C-terminal domain that modulates receptor interaction due to the inclusion of the alternative exon 9b during mRNA splicing. This alteration prevents normal activation of *VEGFR2*-mediated pathways essential for angiogenic signaling, thereby positioning *VEGF165b* as a competitive inhibitor of its pro-angiogenic relatives. It serves as a critical natural antagonist in the angiogenic balance by binding to VEGF receptors without initiating the angiogenic signaling cascade [33]. It is noteworthy that *VEGF165b* binds to *VEGF* receptors with similar affinity but does not trigger angiogenic signaling pathways, making it a natural antagonist of the pro-angiogenic *VEGF* family. This unique feature positions *VEGF165b* as a promising target for anti-angiogenic therapy, particularly in diseases where angiogenesis plays a crucial role, such as HNSCC (Table 1) [27].

In addition to *VEGF165b*, the *VEGF* family includes several other isoforms, such as *VEGF121b*, which also exhibit anti-angiogenic properties. *VEGF121b*, like *VEGF165b*, is a splice variant of *VEGF-A* that plays a significant role in inhibiting angiogenesis and tumor progression. This variant is characterized by its shorter sequence, which lacks the heparin-binding domain, allowing it to remain more diffusible in the extracellular matrix [34].

Recent studies have shown that *VEGF121b* can effectively bind to VEGF receptors (*VEGFR1* and *VEGFR2*) without triggering the pro-angiogenic signaling pathways typically activated by other *VEGF-A* isoforms. Bates et al. (2002) demonstrated that *VEGF165b*, an inhibitory splice variant of *VEGF-A*, binds to *VEGFR2* with an affinity comparable to *VEGF165* but does not induce the phosphorylation required for downstream signaling, thereby inhibiting angiogenesis in vivo. Given the structural similarities, *VEGF121b* is believed to function similarly and has been observed to suppress tumor growth in various cancer models, highlighting its potential as a therapeutic agent [35].

The inclusion of *VEGF121b* in the broader context of anti-angiogenic *VEGF* isoforms underscores the diversity and complexity of the *VEGF* signaling network. By understanding the distinct roles of these isoforms, particularly in contrast to their pro-angiogenic counterparts, researchers can better understand the potential for developing targeted therapies that activate the natural inhibitory mechanisms of *VEGF121b* and *VEGF165b*. The exploration of these isoforms, therefore, opens new avenues for personalized medicine, where the specific angiogenic profile of a tumor could guide the choice of therapeutic intervention. Targeting *VEGF* isoforms such as *VEGF-B*, which can both suppress and facilitate angiogenesis depending on the physiological environment, represents a nuanced approach to anti-angiogenic therapy [36].

The family of *VEGF* splice variants is not random, but a carefully organized process influenced by various physiological and pathological stimuli. For example, hypoxia, a common feature of the tumor microenvironment, activates hypoxia-inducible factor-1 (HIF-1), which in turn can modulate *VEGF* splicing to favor the production of pro-angiogenic isoforms [37]. In addition, splicing factors, including serine/arginine-rich proteins (SRs) and heterogeneous nuclear ribonucleoproteins (hnRNPs), bind *VEGF* mRNA and directly influence the splicing mechanism, thereby dictating the isoform outcome [38]. The expression and dynamic activity of these splicing factors are fundamental in maintaining the delicate balance between pro-angiogenic and anti-angiogenic isoforms of *VEGF*, thus influencing angiogenic potential.

### 2.2. Molecular Perspective

At the molecular level, the interaction between *VEGF* splice variants and their respective receptors develops a complex network of intracellular signaling events. In the case of pro-angiogenic variants, binding to *VEGFR2* is a critical step that triggers the activation of downstream signaling pathways involving multiple kinases and the induction of genes that control endothelial cell proliferation, migration, and survival [39]. These processes are meticulously fine-tuned to ensure that angiogenesis is promoted in a controlled manner under normal physiological conditions. *VEGFR1* has a higher binding affinity for *VEGF-A* isoforms, including both pro-angiogenic and anti-angiogenic variants, than *VEGFR2*. However, its role in angiogenesis is complex and multifaceted. One of the main functions of *VEGFR1* is to act as a decoy receptor. By binding to high-affinity *VEGF* ligands, *VEGFR1* can retain these molecules, preventing them from interacting with *VEGFR2*, which is the main driver of angiogenic signaling. This decoy function of VEGFR1 serves as a regulatory mechanism that modulates the intensity and duration of *VEGFR2*-mediated angiogenesis. For example, under conditions where excessive angiogenesis would be detrimental, such as in certain cancers, *VEGFR1* may limit the availability of *VEGF* to *VEGFR2*, thereby reducing angiogenic signaling [39].

In addition, *VEGFR1* may also function as a modulator of *VEGFR2* activity. Although *VEGFR1* itself has a weaker tyrosine kinase activity compared to *VEGFR2*, it may influence *VEGFR2* signaling through ligand competition and receptor dimerization. When *VEGF165b* or other anti-angiogenic isoforms bind to *VEGFR1*, this may alter the receptor’s interaction with *VEGFR2*, either by promoting heterodimer formation (*VEGFR1*/*VEGFR2*) or by modulating the availability of *VEGFR2* to its pro-angiogenic ligands. This modulation may lead to altered downstream signaling, affecting processes such as endothelial cell proliferation, migration, and survival, which are essential for angiogenesis [40].

In the context of anti-angiogenic therapies, understanding the interplay between *VEGFR1* and *VEGFR2* is essential. Isoforms such as *VEGF165b*, which can bind to *VEGFR1* without activating angiogenic pathways, may harness this decoy function to more effectively suppress *VEGFR2*-mediated signaling. This interaction emphasizes the therapeutic potential of targeting *VEGFR1* together with *VEGFR2* to achieve more precise modulation of angiogenesis in diseases such as cancer [39]. In cancer, however, this balance is disturbed. HNSCC, like many other solid tumors, takes on the angiogenic capabilities of VEGF to enhance its own vascularization, which is essential for tumor growth and metastasis. Pro-angiogenic variants of *VEGF* are often upregulated in tumor cells, contributing to “angiogenic switching”, a phenomenon in which the normal vasculature is transformed into a dynamic and disorganized network of blood vessels [41].

Anti-angiogenic splice variants, such as *VEGF165b*, provide a natural balance to this process. By binding to *VEGF* receptors with an affinity similar to that of pro-angiogenic variants, but without activating angiogenic signaling cascades, *VEGF165b* exerts an inhibitory effect. It effectively acts as a molecular trap, sequestering receptors and preventing pro-angiogenic variants from inducing their effects on endothelial cells. This unique mechanism highlights the potential of *VEGF165b* as a therapeutic agent, providing a way to undermine the aberrant angiogenesis seen in HNSCC and other cancers [42].

Understanding the splice variant profile of *VEGF* in tumors is important not only for the development of targeted therapies but also for prognostication and treatment planning. While current anti-angiogenic therapies focus primarily on inhibiting the activity of pro-angiogenic *VEGF* variants, resistance to these treatments is not uncommon. This is in part due to the ability of the tumor to adapt by finding alternative pathways to promote angiogenesis or by modulating the expression of *VEGF* variants [43]. More effective therapeutic strategies can be developed by obtaining a deeper insight into the splicing mechanisms and regulatory factors that determine the *VEGF* isoform landscape within a tumor.

Future therapeutic approaches may include novel technologies such as gene editing or splice-switching oligonucleotides that can selectively alter *VEGF* mRNA splicing, thereby increasing the expression of anti-angiogenic variants such as *VEGF165b* [44,45]. Such precision medicine strategies could provide more personalized and effective treatment options for patients with HNSCC, potentially improving outcomes while minimizing side effects.

The multifaceted interaction between different splice forms of *VEGF* represents a complex but promising frontier in cancer biology. The ability to modulate this balance by enhancing natural inhibitors of angiogenesis while reducing pro-angiogenic factors opens the door to innovative treatments for HNSCC. Further research into the regulation, function, and therapeutic targeting of *VEGF* splice variants will undoubtedly continue to shed light on the pathophysiology of angiogenesis and offer new hope to patients struggling with HNSCC.

## 3. Experimental Evidence of *VEGF* Splice Variants in Angiogenesis Inhibition

The study of *VEGF* splice variants has become increasingly important in understanding angiogenesis inhibition, particularly in the context of preclinical studies. Several investigators have explored the role of *VEGF-A* splicing mechanisms and their implications for anti-angiogenic therapeutics. For example, they have highlighted the critical nature of *VEGF-A* splicing in the development of anti-angiogenic therapies, suggesting that manipulation of these splicing events may provide novel therapeutic avenues [46]. Furthermore, the discovery of alternatively spliced *VEGFR-*2 by Albuquerque et al. (2009) as a major endogenous inhibitor of lymphatic vessel growth highlights the complexity and importance of *VEGF* splicing variants in regulating angiogenesis. This receptor variant acts as a critical modulator and provides a potential target for therapeutic intervention in diseases characterized by abnormal lymphangiogenesis [47].

In addition, the interaction between VEGF and other molecular pathways has been the subject of intense research. For example, the *VEGF-A/SOX2/SRSF2* network, as studied by Cherine Abou Faycal et al. (2019), plays a key role in the regulation of alternative splicing of VEGFR1 pre-mRNA in lung cancer cells, illustrating the complex regulatory mechanisms that control angiogenesis in cancer [48]. Furthermore, Moens et al. (2014) discussed the multifaceted activity of *VEGF* in angiogenesis and its implications for therapeutic responses, highlighting the complexity of *VEGF’s* role in promoting angiogenesis and how this may affect the efficacy of therapeutic strategies [49].

The study of *VEGF* splicing variants in angiogenesis inhibition is important not only for understanding the basic mechanisms of angiogenesis but also for developing targeted therapies that can effectively modulate these pathways to treat a variety of diseases, including cancer and vascular disorders.

### 3.1. Preclinical Studies

Over the past decade, preclinical studies have extensively explored the potential of *VEGF165* as a therapeutic agent in the treatment of HNSCC. An important study by Zhang et al. (2018) introduced a *VEGF165b* variant with improved half-life and superior antitumor potency in a mouse model, demonstrating the potential for improved therapeutic interventions in cancer treatment. This variant exhibited extended stability and enhanced efficacy in inhibiting tumor angiogenesis, highlighting the value of targeting *VEGF* pathways in cancer therapy [50].

The interaction between *VEGF165* and other molecular pathways has also been of interest. For example, Kim et al. (2017) explored how *VEGFA* links self-renewal and metastasis through the induction of Sox2 to repress miR-452 and Slug stimulation, providing insight into mechanisms to exploit *VEGF165* therapeutically [51]. Additionally, research efforts, such as those by Koyama et al. (2017), have attempted to normalize tumor vasculature by targeting *VEGF* pathways with the goal of restoring chemotherapeutic sensitivity in cancer models. This approach highlights the therapeutic potential of modulating *VEGF165* expression and activity to improve treatment outcomes [52].

These and other studies in the field continue to provide valuable insights into the role of VEGF165 in cancer biology and its potential as a target for therapeutic intervention. By understanding the mechanisms by which *VEGF165* affects angiogenesis and tumor growth, researchers are developing more effective strategies to combat HNSCC and other cancers.

### 3.2. Clinical Perspectives on VEGF165 as a Treatment for HNSCC

In recent years, clinical research on *VEGF165* has gained significant attention, particularly in its application as a therapeutic target in HNSCC. *VEGF165* has been the focus of various studies aimed at inhibiting tumor growth and metastasis by modulating blood vessel formation. A notable study by Vassilakopoulou et al. (2015) focused on targeting angiogenesis in HNSCC, emphasizing the potential of *VEGF165* inhibitors to improve treatment outcomes for patients with this aggressive cancer. This research underscores the importance of angiogenesis in cancer progression and the therapeutic benefits of inhibiting the *VEGF* pathway [53]. In the field of differentiated thyroid carcinoma (DTC), Abdel Rahman (2015) explored the efficacy of targeting the *VEGF* pathway in iodine-refractory DTC, demonstrating the potential of VEGF165 inhibitors to provide a therapeutic advantage in cases where conventional treatments are insufficient [54].

Neufeld et al.’s (1999) comprehensive review of *VEGF* and its receptors further elucidates the mechanistic basis of *VEGF*-mediated angiogenesis, providing insight into how clinical interventions targeting *VEGF165* could be optimized to enhance antitumor effects while minimizing adverse outcomes [55]. In addition, Ashina et al. (2015) provided evidence that *VEGF*-induced increases in blood flow cause vascular hyperpermeability in vivo, contributing to our understanding of how modulation of VEGF165 may impact the tumor microenvironment and treatment efficacy [56].

These clinical studies highlight the therapeutic potential of targeting VEGF165 in the treatment of cancer and provide new avenues for the development of effective anti-angiogenic therapies. By inhibiting *VEGF*-mediated pathways, researchers aim to deprive tumors of blood supply and thereby inhibit growth and metastasis. As our understanding of the role of *VEGF165* in cancer biology deepens, new therapeutic strategies are expected to emerge and improve outcomes for patients with HNSCC and other cancers.

### 3.3. Potential Limitations and Future Directions

**Figure 2:**
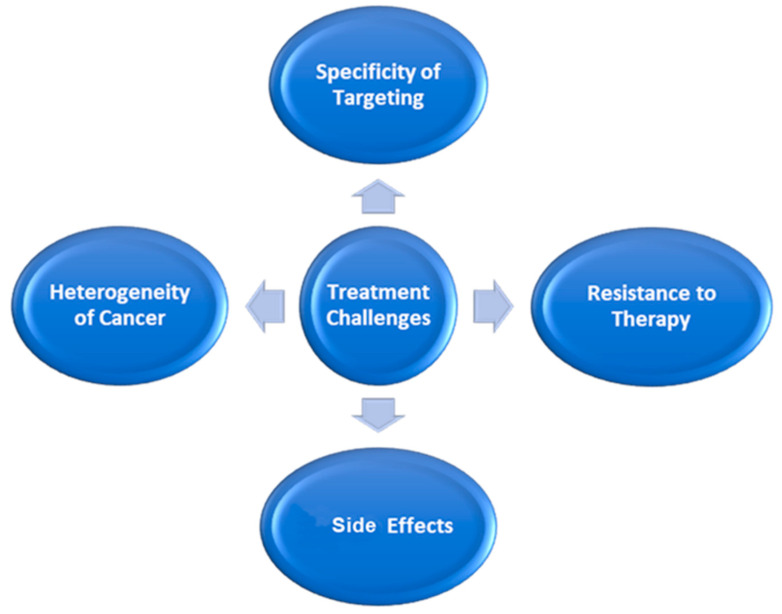
Focusing on the key issues of target specificity, each element represents a critical factor influencing the efficacy and outcome of cancer treatments.

Therapeutic targeting of *VEGF* splice variants for the treatment of cancer and other diseases represents a promising avenue for medical research and patient care. However, this approach is not without limitations and challenges (Figure 2). The complexity of *VEGF* signaling and its indispensable role in physiological and pathological angiogenesis requires a nuanced understanding of the potential obstacles to the development of effective therapies [33].

One of the major challenges in targeting *VEGF* splice variants is the specificity of therapeutic agents. *VEGF* and its variants are involved in both healthy and pathologic states, contributing to wound healing, normal vascular function, and pathologic angiogenesis [57]. Developing therapies that selectively inhibit the pathological functions of *VEGF* splice variants without interfering with their physiological roles remains a significant challenge. As with many targeted cancer therapies, there is a potential for resistance to treatments targeting *VEGF* splice variants. Tumors may be able to activate alternative angiogenic pathways or mechanisms to circumvent the inhibition of VEGF signaling, and thus reduce the efficacy of these targeted therapies over time [58,59].

Targeting *VEGF* splice variants may result in adverse effects due to the inhibition of normal angiogenesis and vascular maintenance. Potential side effects include impaired wound healing, hypertension, proteinuria, and increased risk of thromboembolic events. These risks require careful consideration and management in the clinical setting [60]. The heterogeneity of tumors is another challenge. Expression levels and roles of *VEGF* splice variants can vary significantly between cancers and within tumors of the same type. This variability may influence the responsiveness of tumors to *VEGF*-targeted therapies and complicate the development of generally effective treatments [61].

Efficient delivery of therapeutic agents targeting *VEGF* splice variants to the tumor site is critical to their success. However, abnormal and often inefficient tumor vasculature can impede the path and distribution of these agents, limiting their therapeutic potential [62]. The development of therapies targeting *VEGF* splice variants also faces regulatory and ethical considerations. Rigorous clinical trials are required to demonstrate the safety and efficacy of these treatments and the high costs associated with research and development may impact the accessibility and affordability of approved therapies [63].

Despite these challenges, the limitations associated with targeting *VEGF* splice variants continue to be addressed through continued research and technological advances. To overcome these obstacles and improve the efficacy of treatments for cancer and other angiogenesis-related diseases, strategies such as combination therapies, advanced drug delivery systems, and personalized medicine approaches are being explored.

## 4. Pro-Angiogenic vs. Anti-Angiogenic *VEGF* Variants in HNSCC: Clinical Impact

VEGF has long been recognized as an essential regulator of angiogenesis and plays a key role in carcinoma progression. This complex role is further complicated by the existence of *VEGF* splice variants that can promote (pro-angiogenic) or inhibit (anti-angiogenic) angiogenesis, thereby influencing the tumor microenvironment and response to therapy. While pro-angiogenic variants, such as *VEGF-A*, are well studied for their role in facilitating tumor growth and metastasis by promoting the formation of new blood vessels, anti-angiogenic splice variants, represented by VEGF165b, provide a natural countermeasure by inhibiting these processes [39]. This section aims to outline the clinical implications of these opposing forces in the context of HNSCC, setting the stage for a discussion of how utilizing the balance between pro-angiogenic and anti-angiogenic *VEGF* variants may provide new avenues for targeted therapies. The intricate interplay between these variants not only underscores the complexity of tumor angiogenesis but also demonstrates the potential for personalized treatment strategies that could improve patient outcomes in HNSCC.

In the field of HNSCC, the dichotomy between pro- and anti-angiogenic *VEGF* variants is emerging as an essential aspect of tumor biology and therapeutic targeting. In particular, the *VEGF165* variant exemplifies the pro-angiogenic group, which promotes tumor growth and metastasis by increasing angiogenesis. This variant, by binding to *VEGFR2*, triggers an intracellular signaling cascade that culminates in endothelial cell proliferation, migration, and new blood vessel formation, thereby facilitating tumor progression [64].

In contrast, *VEGF165b*, a splice variant of *VEGF-A*, is unique among anti-angiogenic factors in its ability to bind to *VEGF* receptors without activation of downstream pro-angiogenic pathways. This interaction effectively blocks angiogenic signals that would otherwise be mediated by pro-angiogenic variants, illustrating a natural mechanism of angiogenesis inhibition in the tumor microenvironment [65]. The implications of these opposing functions are profound, affecting not only cancer progression but also the efficacy and development of anti-angiogenic therapies.

Recent studies have revealed the complex role these variants play in cancer. For example, research has shown that altering the balance between pro- and anti-angiogenic *VEGF* variants can have a significant impact on disease progression and response to treatment. Therapies that shift this balance toward anti-angiogenesis have shown promise in preclinical models of HNSCC, suggesting a potential pathway for the development of novel treatment strategies that exploit the inherent anti-angiogenic properties of variants such as *VEGF165b* [66].

In this complicated context of tumor angiogenesis, particularly in HNSCC, the distinction between pro-angiogenic and anti-angiogenic *VEGF* variants provides crucial insight into therapeutic targeting and disease progression. Table 2 below consolidates these variants, delineating their distinct roles, clinical relevance, and basis for further investigation, thus providing a foundation for understanding the potential for targeted modulation of angiogenesis in HNSCC therapy.

The balance between pro- and anti-angiogenic variants of *VEGF* plays an important role in the angiogenic landscape of head and neck squamous cell carcinoma. Pro-angiogenic variants, particularly *VEGF-A*, facilitate tumor progression by promoting vascular permeability and endothelial cell proliferation, leading to the formation of new blood vessels [58]. In contrast, anti-angiogenic variants, such as *VEGF165b*, act as natural inhibitors of angiogenesis, potentially limiting tumor growth and spread by binding to *VEGF* receptors without activating downstream pro-angiogenic pathways [74].

Therapeutic manipulation of this balance to suppress tumor angiogenesis while promoting the body’s natural anti-angiogenic mechanisms is a promising approach to the treatment of HNSCC. The targeting of *VEGF*-specific signaling pathways with monoclonal antibodies or tyrosine kinase inhibitors has been shown to be effective in reducing vascularization and tumor growth. However, developing resistance to these agents requires a deeper understanding of the complex interplay between *VEGF* variants and the tumor microenvironment [75].

The clinical implications of the pro- and anti-angiogenic balance of *VEGF* variants are profound. The efficacy of existing therapies, in particular anti-angiogenic drugs, can be significantly influenced by this balance, with an imbalance in favor of the pro-angiogenic variants being associated with aggressive tumor growth and a poorer outcome in patients. Resistance to anti-angiogenic therapy, which is a major challenge in the treatment of HNSCC, is often due to tumor adaptation strategies, such as the increase in the number of alternative angiogenic factors or receptors, which render the treatments ineffective over time [50].

Personalizing therapeutic approaches based on each patient’s *VEGF* variant profile could improve treatment efficacy and overcome resistance. Biomarker-based strategies, including quantifying pro- and anti-angiogenic *VEGF* variants in tumor samples or circulating blood, offer a potential method for more effectively personalizing therapy. Such personalized interventions could improve patient response and minimize side effects by avoiding unnecessary broad-spectrum anti-angiogenic exposure [76].

Moving forward, the development of next-generation therapies for HNSCC will depend on advances in understanding the biology of *VEGF* and the mechanisms underlying resistance to anti-angiogenic therapy. Elucidating the precise roles of different *VEGF* variants in tumor angiogenesis and exploring novel therapeutic targets within these pathways should be the focus of future research. Additionally, the implementation of advanced diagnostic techniques to assess *VEGF* variant profiles promises to refine treatment strategies, paving the way for more effective and durable HNSCC responses.

## 5. Resistance Mechanisms and Overcoming Therapeutic Challenges

A major advance in the field of oncology has been the development of anti-angiogenic therapies targeting *VEGF* splice variants in the context of cancer treatment. However, the emergence of resistance to these therapies represents a significant challenge, requiring a deeper understanding of the underlying mechanisms and the exploration of strategies to overcome these barriers, such as a detailed examination of how tumors can avoid *VEGF* blockade by activating other angiogenic pathways, such as those mediated by fibroblast growth factors (FGFs) or angiopoietins. This compensatory mechanism undermines the efficacy of *VEGF*-targeted treatments and is a major obstacle in cancer therapy [77].

It is important to consider vascular co-option, which is a process by which tumors make use of existing blood vessels rather than forming new ones. This mechanism of resistance to anti-angiogenic therapy is particularly prevalent in liver metastases and presents a challenge to the efficacy of *VEGF*-targeted approaches [78]. The tumor microenvironment also has an important role in resistance. Further complicating the therapeutic landscape, tumor hypoxia can induce the expression of other pro-angiogenic factors. Immune cells in the tumor microenvironment can create immunosuppressive environments supporting tumor growth and angiogenesis [79]. A multifaceted approach is needed to overcome these challenges. It requires not only understanding the underlying mechanisms of resistance but also developing strategies to counter these mechanisms. This could include combination therapies that target multiple angiogenic pathways, modulate the tumor microenvironment, or enhance the immune response against tumor cells.

Research has shown that the diversity of *VEGF* splice variants provides new insights for targeted therapy. Montemagno et al. (2023) demonstrated that novel *VEGF* splice variants in renal cell carcinoma are less effectively inhibited by conventional anti-*VEGF/VEGFR* therapies, suggesting that these variants may serve as alternative therapeutic targets for patients resistant to current treatments. Their study highlights the importance of understanding the variability in the expression of *VEGF* splice variants and how it affects patient-specific treatment strategies [59].

Subsequent breast cancer research has shown that *VEGF-A* and its splice variants significantly influence tumor development, with clinical implications that could potentially refine therapeutic approaches. According to Kawas et al. (2022), the roles of these splice variants provide a deeper understanding of their involvement in breast cancer progression and a basis for the development of splice variant-specific therapies. This approach could enhance the efficacy of anti-angiogenic treatments and improve patient outcomes [80].

A further level of complexity in the approach to angiogenesis is introduced by the different binding affinities of *VEGF-A* splice variants to *VEGF* receptors (VEGFR). Mamer et al. (2020) showed that these variants bind *VEGFR* with different affinities and thus differentially affect angiogenic signaling pathways. This finding paves the way for designing more precise anti-angiogenic agents that selectively inhibit specific *VEGF/VEGFR* interactions, potentially reducing side effects and increasing therapeutic efficacy [81].

The appearance of *VEGF165b*, a splice variant of *VEGF-A*, was particularly remarkable. Boudria et al. (2018) demonstrated its role in the promotion of lung tumor progression and resistance to anti-angiogenic therapies. *VEGF165b* functions through an autocrine β1 integrin/VEGFR feedback loop, increasing tumor aggressiveness and circumventing the effects of current anti-angiogenic drugs. Targeting this splice variant may provide a novel strategy to overcome resistance and achieve better control of tumor progression [33]. Prince et al. (2019) shed light on novel approaches to inhibit *VEGF165*-mediated angiogenic pathways, offering hope for more effective treatments for HNSCC. For example, adjuvant anti-angiogenic therapy targeting *VEGFR2* and *VEGFR3* has shown promise in improving chemotherapeutic uptake in HNSCC models. The goal of this approach is to inhibit tumor vascularization and therefore improve the delivery and efficacy of chemotherapeutic agents [82].

Furthermore, the combination of anti-PD-1 monoclonal antibodies with anti-VEGF agents has emerged as a safe and effective strategy for the second-line or subsequent therapy of recurrent or metastatic HNSCC. This combination therapy not only directly targets tumor cells, but also disrupts the blood supply to the tumor. This provides a multifaceted attack against the cancer. The importance of *VEGF* and its splice variants in the pathogenesis and treatment of HNSCC is underscored by the study of angiogenesis and anti-angiogenic therapy in HNSCC [83]. The understanding of the specific roles and mechanisms of action of *VEGF165* in tumor angiogenesis will provide a solid foundation for the development of targeted therapies that could significantly improve the outcome of patients with HNSCC.

The outcome would underscore the importance of understanding the mechanisms of resistance to therapies targeting *VEGF* and the need to develop multifaceted strategies to overcome these challenges. This would underscore the continued need for research to identify new targets, understand the tumor microenvironment, and tailor treatments to individual patient profiles to improve outcomes in cancer therapy.

## 6. Conclusions

In conclusion, this review presents the importance of *VEGF* splice variants, in particular *VEGF165b*, in inhibiting angiogenesis in the context of head and neck squamous cell carcinoma. *VEGF165b* is a promising therapeutic target due to its unique anti-angiogenic properties and its divergence from the pro-angiogenic activities typically attributed to *VEGF* isoforms. Further exploration of *VEGF* splice variants as tools for personalized medicine is essential and may reshape the management of HNSCC. This review also recognizes the challenges facing the *VEGF* splice variant approach, including target specificity, resistance to therapy, adverse effects, cancer heterogeneity, and regulatory and ethical considerations.

## Figures and Tables

**Table 1:** The table indicates that while *VEGF121*, *VEGF165*, and *VEGF189* are pro-angiogenic, promoting the formation of new blood vessels, *VEGF165b* is anti-angiogenic, inhibiting the angiogenic pathways.

VEGF Splice	Variant	Receptor Binding	Biological Activity	Role in Angiogenesis
*VEGF121*	Binds	*VEGFR1* and *VEGFR2*	Initiates cellular events leading to new vessel formation	Pro-angiogenic
*VEGF165*	Binds	*VEGFR1* and *VEGFR2*	Promotes proliferation and migration of endothelial cells	Pro-angiogenic
*VEGF189*	Binds	*VEGFR1* and *VEGFR2*	Influences extracellular matrix affecting vascular growth	Pro-angiogenic
*VEGF165b*	Binds	*VEGF* receptors without activating angiogenic signaling	Inhibits angiogenic pathways, acts as a competitive inhibitor	Anti-angiogenic

**Table 2:** Comparative analysis of *VEGF* variants in angiogenesis, detailing their pro-angiogenic and anti-angiogenic actions.

VEGF Variant	Type (Pro-Angiogenic/Anti-Angiogenic)	Characteristics	Clinical Implications
*VEGF-A165* [67]	Pro-angiogenic	Promotes endothelial cell proliferation, migration, and new blood vessel formation by binding to *VEGFR1* and *VEGFR2*.	Associated with tumor progression and metastasis in various cancers, including HNSCC.
*VEGF-A121* [68]	Pro-angiogenic	Similar to *VEGF-A165* but more diffusible due to the lack of heparin-binding domains.	Plays a role in angiogenesis and tumor growth.
*VEGF-A189* [69]	Pro-angiogenic	Strongly binds to heparin and extracellular matrix components, affecting local angiogenesis.	Influences the angiogenic profile in specific tissue environments.
*VEGF-A165b* [70]	Anti-angiogenic	A splice variant of *VEGF-A165* that binds to *VEGFR1* and *VEGFR2* without activating pro-angiogenic signaling pathways.	Inhibits angiogenesis, offering a potential therapeutic target for reducing tumor growth and angiogenesis in cancers.
*VEGF-A121b* [9]	Anti-angiogenic	A splice variant of *VEGF-A121* that also inhibits angiogenesis by preventing *VEGFR*-mediated signaling.	Potentially reduces angiogenesis and tumor progression, similar to *VEGF-A165b.*
*VEGF-B* [35]	Pro-angiogenic	Binds primarily to *VEGFR1*, involved in heart development and fatty acid uptake.	Role in cancer is less clear but may be involved in metabolic regulation and survival of cancer cells.
*VEGF-C* [71]	Pro-angiogenic	Induces lymphangiogenesis and angiogenesis through binding to *VEGFR2* and *VEGFR3*.	Implicated in lymphatic metastasis of solid tumors, including HNSCC, by promoting lymphangiogenesis.
*VEGF-D* [72]	Pro-angiogenic	Similar to *VEGF-C*, promotes lymphangiogenesis and angiogenesis by binding to *VEGFR2* and *VEGFR3*.	Potential role in lymphatic spread and metastasis of cancer, including implications for HNSCC.
*PIGF* [73]	Pro-angiogenic	Binds to VEGFR1 and NRP1; involved in pathological angiogenesis, inflammation, and recruitment of myeloid cells.	Studied for its potential in cancer therapy and cardiovascular diseases, though with varying implications in different types of cancer.

## Data Availability

No new data were created or analyzed in this study. Data sharing is not applicable to this article.
